# The dual function of PRMT1 in modulating epithelial-mesenchymal transition and cellular senescence in breast cancer cells through regulation of ZEB1

**DOI:** 10.1038/srep19874

**Published:** 2016-01-27

**Authors:** Yanyan Gao, Yaping Zhao, Juechao Zhang, Yang Lu, Xin Liu, Pengyu Geng, Baiqu Huang, Yu Zhang, Jun Lu

**Affiliations:** 1 The Institute of Genetics and Cytology, Northeast Normal University, Changchun 130024, China; 2 The Key Laboratory of Molecular Epigenetics of the Ministry of Education, Northeast Normal University, Changchun 130021, China

## Abstract

Although the involvement of protein arginine methyltransferase 1 (PRMT1) in tumorigenesis has been reported, its roles in breast cancer progression and metastasis has not been elucidated. Here we identified PRMT1 as a key regulator of the epithelial-mesenchymal transition (EMT) in breast cancer. We showed that the EMT program induced by PRMT1 endowed the human mammary epithelial cells with cancer stem cell properties. Moreover, PRMT1 promoted the migratory and invasive behaviors in breast cancer cells. We also demonstrated that abrogation of PRMT1 expression in breast cancer cells abated metastasis *in vivo* in mouse model. In addition, knockdown of PRMT1 arrested cell growth in G1 tetraploidy and induced cellular senescence. Mechanistically, PRMT1 impacted EMT process and cellular senescence by mediating the asymmetric dimethylation of arginine 3 of histone H4 (H4R3me2as) at the ZEB1 promoter to activate its transcription, indicating the essential roles of this epigenetic control both in EMT and in senescence. Thus, we unraveled a dual function of PRMT1 in modulation of both EMT and senescence *via* regulating ZEB1. This finding points to the potent value of PRMT1 as a dual therapeutic target for preventing metastasis and for inhibiting cancer cell growth in malignant breast cancer patients.

Breast cancer is the most common diagnosed cancer and a leading cause of cancer-related death in Chinese women^1^. The major cause of fatality in breast cancer is distant metastases. The epithelial-to-mesenchymal transition (EMT) is a cellular process in which epithelial cells lose their cell-cell adhesion and polarized organization, and acquire the spindle-like morphology with enhanced cell migration and invasion. Although EMT was first described in embryogenesis and development^2^, it is increasingly accepted as a crucial step for tumour infiltration and metastasis^3^. Recent studies investigating the *in vivo* relevance of EMT to tumour metastasis revealed that the EMT-like features were enriched both in mammary metastatic models of mice and in clinical breast cancer samples^4^,^5^,^6^,^7^. These studies provided convincing support for the actual role of EMT in breast cancer metastasis. Therefore, targeting EMT in breast cancer has been evaluated as an important therapeutic intervention^8^.

In contrast to EMT program, cellular senescence has been proposed as a crucial tumour-suppressive mechanism that causes irreversible cell cycle arrest against the initiation and progression of cancer. Increasing evidence suggests that several EMT-inducing transcription factors convey the cancer cells the potentiality to avoid senescence^9^. For instance, expression of Twist1 was shown to overcome the oncogene-induced senescence, whereas it can promote tumour initiation and metastasis in breast and lung cancer models^10^,^11^. Both ZEB1 and ZEB2 were sufficient to suppress the oncogene-induced senescence triggered by overexpression of EGFR (epidermal growth factor receptor) in human oesophageal epithelial cells^12^. Contrarily, deletion of Twist1, Snail1 or ZEB1, respectively, induced senescence in murine breast cancer cells^10^, human prostate cancer cell lines^13^, and murine embryonic fibroblasts^14^. The general mechanisms by which these EMT-associated transcription factors act in senescence remain to be elucidated, yet several key cyclin-dependent kinase inhibitors, such as p16^INK4A^, p15^INK4B^, p19^ARF^ and p21^WAF1^ were shown to be regulated as subsequent events to modulate senescence^10^,^13^,^14^. The capacity to inhibit such tumour failsafe programs as senescence and apoptosis seems to be a common property of EMT-induced factors. Theoretically, new therapeutic strategies that targeting the major players promoting EMT simultaneously inhibiting senescence have a double impact, i.e., preventing tumour dissemination in metastatic lesions while eradicating existing metastatic cancer cells. Hence, better understanding towards the regulation of EMT and senescence will shed light on our cancer therapeutic strategies.

The protein arginine methyltransferases (PRMTs), a family of enzymes catalyzing arginine methylation, have been shown to be able to methylate a variety of protein substrates^15^ to influence many cellular processes, including RNA processing, gene transcription, DNA damage repair, signal transduction and protein translocation^16^. PRMT1 is a predominant asymmetric arginine methyltransferase in human, and it responses for abundant protein substrates, such as FOXO1, ERα, MRE11, 53BP1 and histone H4^17^,^18^,^19^,^20^,^21^. Asymmetric dimethylation of histone H4 at arginine 3 (H4R3me2as) mediated by PRMT1 is a critical modification for active chromatin^22^. Increasing evidence has linked PRMT1 to the development and progression of cancers. Aberrant expression of PRMT1 has been observed in several cancers, including breast cancer, lung cancer, colon cancer, bladder cancer, acute myeloid leukemia and mixed lineage leukemia^23^. PRMT1 is an essential component of MLL oncogenic complexes, and the H4R3me2as modification has a critical function in the expression of MLL downstream targets^24^. Interestingly, high expression of PRMT1 has shown to be indicative of the disease progression and aggressiveness in breast and colon cancer^25^,^26^. Moreover, H4R3me2as was found to be positively correlated with increasing tumour grade in prostate cancer^27^. These findings indicate that both PRMT1 and H4R3me2as may probably contribute to tumour malignancy and aggressiveness. However, the mechanism how PRMT1 is involved in tumorigenesis and metastasis remains unknown.

In this study, we demonstrated that PRMT1 was able to induce the EMT process and to enhance the capabilities of migration and invasion in breast cancer cells. Besides, PRMT1 dramatically increased the population of stem-like cells in human mammary epithelial cells. Meanwhile, knockdown of PRMT1 not only suppressed metastasis *in vivo* in mice, but also provoked cellular senescence in breast cancer cells. These functional effects of PRMT1 were exerted through the control of ZEB1 transcriptional expression *via* H4R3me2as modification at gene’s promoter. Thus, we have identified a novel role and regulatory mechanism of PRMT1 in breast cancer cell proliferation and metastasis, which may provide clues for the development of new epigenetic intervention targeting PRMT1 for advanced breast cancers.

## Results

### PRMT1 was able to bring about the characteristics of EMT in breast cancer cells

**Figure 1:**
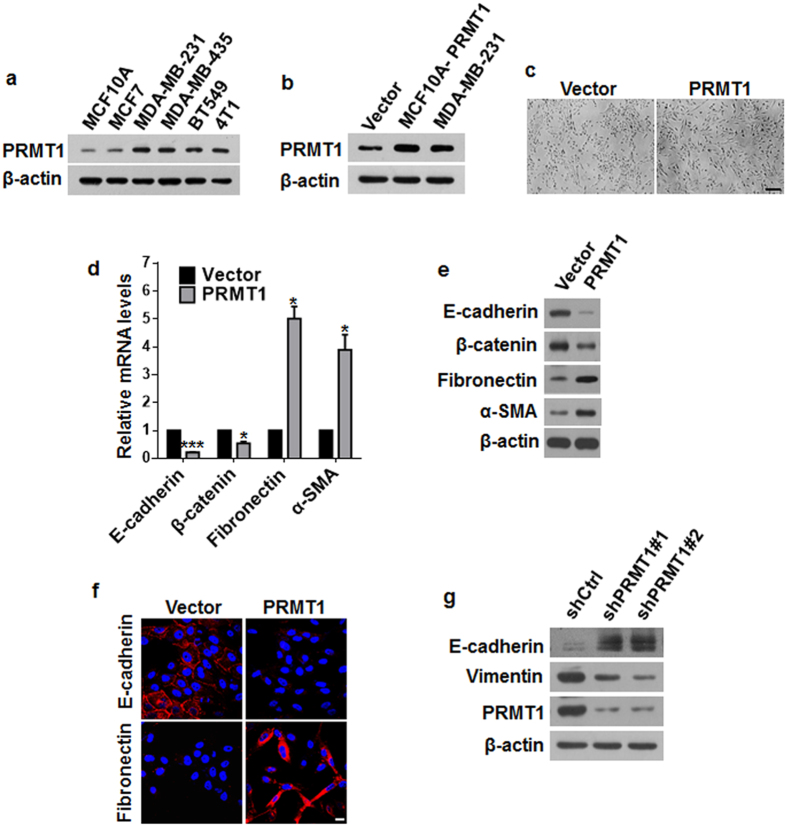
PRMT1 induced epithelial-mesenchymal transition in breast cancer cells. (**a**) PRMT1 expression in different human breast cancer cell lines was analyzed by western blotting. (**b**) Overexpression of PRMT1 in stable lentivirus infected MCF10A cells, and endogenous expression of PRMT1 in MDA-MB-231 were confirmed by western blotting. (**c**) Representative phase-contrast images of morphologic changes in MCF10A-PRMT1 and MCF10A-vector cells. Scale bars: 200 μm. (**d–f**) Expression of epithelial and mesenchymal markers was analyzed by immunofluorescence (**f**), western blotting (**e**), and qRT-PCR (**d**). Scale bars: 10 μm in the inset of (**f**). (**g**) Western blots of PRMT1, epithelial and mesenchymal marker expression in MDA-MB-231-shCtrl, shPRMT1#1/#2 cells after lentivirus infection. All experiments were repeated three times. Error bars, mean ± SD, *P < 0.05, ***P < 0.001.

To determine the correlation of PRMT1 expression level with the malignancy and metastasis of breast cancer cells, we first compared the PRMT1 level in normal mammary epithelial MCF10A cell and multiple breast cancer cell lines. Western blotting revealed that the PRMT1 expression was apparently higher in cell lines known to be highly metastatic (MDA-MB-231, MDA-MB-435, BT549 and 4T1) than that in breast epithelia cancer cells (MCF7) and normal mammary epithelial cells (MCF10A) (Fig. 1a). We then explored whether PRMT1 can trigger EMT in breast cancer cells. We stably overexpressed PRMT1 in MCF10A cells (designated MCF10A-PRMT1) by using lentivirus infection, in which the PRMT1 expression level was comparable to the endogenous PRMT1 expression in MDA-MB-231 (Fig. 1b). We found that the MCF10A-PRMT1 cells displayed a dramatic change in cell morphology, characterized by the transformation from the cobblestone-like epithelial cells with tight cell-to-cell adhesion, into a spindle-shaped fibroblast-like morphology with distinct cellular scattering (Fig. 1c). At the molecular level, the MCF10A-PRMT1 cells exhibited a prominent upregulation of the mesenchymal markers fibronectin and alpha-smooth muscle actin (α-SMA), with a simultaneous downregulation of the epithelial markers E-cadherin and β-catenin, at both mRNA and protein levels (Fig. 1d,e). Similar results were observed in breast epithelia cancer cell MCF7-PRMT1 (Supporting Information Fig. S1a,1b). These changes in molecular markers were further confirmed by inspecting the subcellular localization of the proteins using immunofluorescence staining. As can be seen, MCF10A-PRMT1 cells showed a loss of E-cadherin membranous cell-cell junction staining, while the mesenchymal marker fibronectin was strongly stained (Fig. 1f). In addition, a loss-of-function study in a breast cancer cell line MDA-MB-231 that expresses PRMT1 at a detectable level was performed. The results showed that knockdown of PRMT1 in MDA-MB-231 cells (MDA-MB-231-shPRMT1) augmented the expression of E-cadherin and reduced the expression of vimentin (Fig. 1g). Together, these results implicate that PRMT1 is a novel regulator of EMT in breast cancer cells.

### PRMT1 promoted the migration and invasion in breast cancer cells

**Figure 2:**
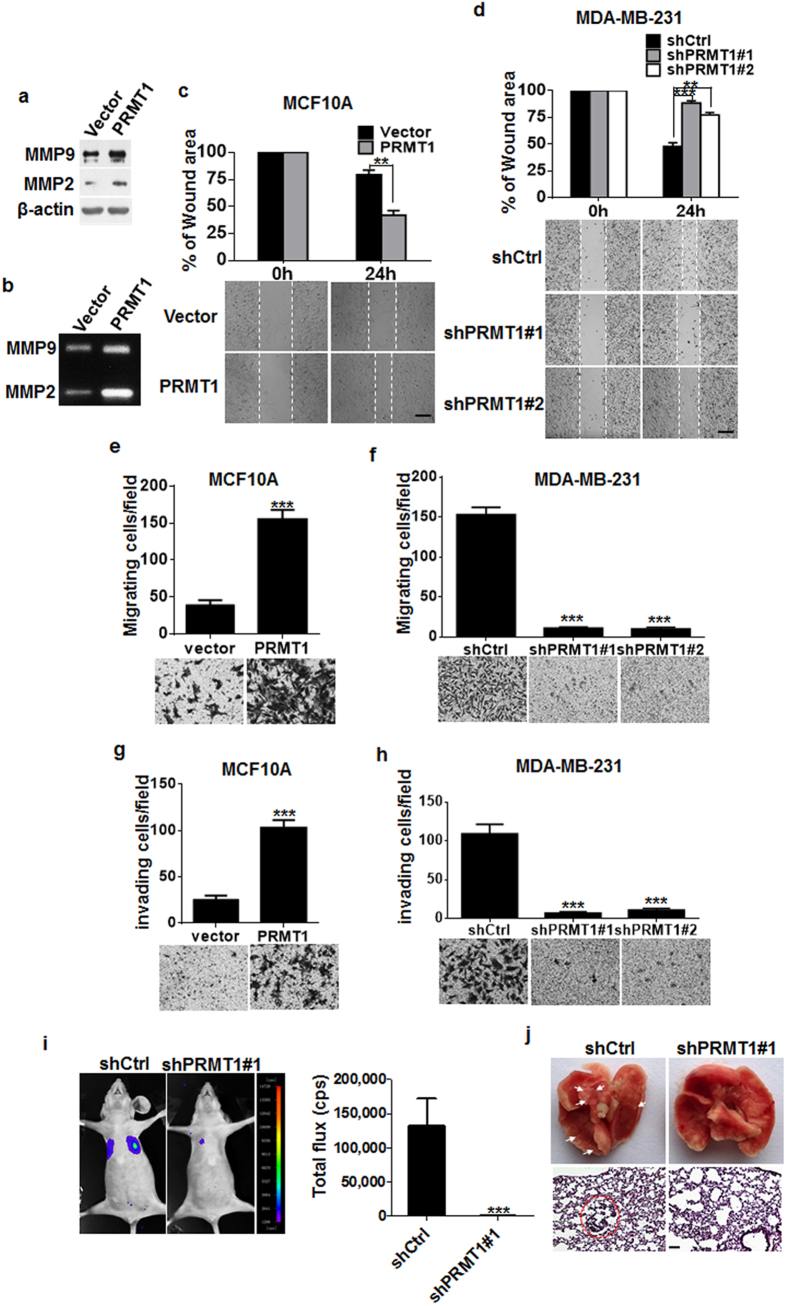
PRMT1 promoted migration and invasion in breast cancer cells. (**a**) Western blotting analyses of MMP2 and MMP9 expression in MCF10A-PRMT1 and in control vector cells. (**b**) Assessment of the MMP2 and MMP9 enzymatic activities by gelatin zymography in MCF10A-PRMT1 and in control vector cells. (**c,d**) Representative images from the wound healing assays in MCF10A-PRMT1 and in MDA-MB-231-shPRMT1#1/#2 cells. Scale bars: 200 μm. Top panel represents statistical analyses of wound-healing assays, performed as described in the Materials and methods. (**e,f**) Migration assays in MCF10A-PRMT1 and MDA-MB-231-shPRMT1#1/#2 cells. (**g–h**) Invasion assays in MCF10A-PRMT1 and MDA-MB-231-shPRMT7#1/#2 cells. Representative images of migrated and invaded cells are shown. Numbers of cells that penetrated through the Transwell chambers in the absence (**e**,**f**: migrating cells/field) or presence (**g**,**h**: invading cells/field) of Matrigel were determined. Top panel represents the mean number of cells per field. Cells from five randomly chosen fields were counted, and each experiment was repeated at least three times. (**i**) Representative bioluminescence images of lung metastases in mice via tail vein injection of indicated cells, and the metastases were quantified by measuring the photo flux (mean of 5 mice). (**j**) Visible lung metastatic nodules are represented in the graph. Representative H&E stained lung sections are displayed in the lower panel. Scale bars: 100 μm. Error bars, mean ± SD, **P < 0.01, ***P < 0.001.

The EMT progress is typically accompanied by the loss of the cell-cell contacts and the acquisition of migratory and invasive properties. We next examined if PRMT1 can affect the migration and invasion in breast cancer cells. We showed that ectopic expression of PRMT1 led to increased MMP2 and MMP9 expression (Fig. 2a). Moreover, the activities of mature proenzymes of MMP2 and MMP9 upon cleavage were significantly augmented as measured by gelatin zymography (Fig. 2b). The effect of PRMT1 on cell migration and invasion was assessed by wound healing assay and transwell chamber assay in culture media lacking serum to exclude the effect of cell proliferation. The results demonstrated that PRMT1 overexpression remarkably encouraged the MCF10A migration ability (Fig. 2c,e), whereas knockdown of PRMT1 decreased the migration of MDA-MB-231 cells (Fig. 2d,f). Similarly, the invasion assay confirmed that overexpression of PRMT1 promoted the invasion capacity (Fig. 2g), while loss of PRMT1 reduced the invasion capacity in MDA-MB-231 cells (Fig. 2h).

To verify the *in vitro* observations, we investigated the effect of PRMT1 on the distant metastasis of breast cancer cells *in vivo* in nude mice. MDA-MB-231-shPRMT1#1 cells or non-targeting control shRNA were injected into the lateral tail veins of female nude mice, and the metastatic lung tumours were examined. Strikingly, we found that the mice injected with MDA-MB-231-shPRMT1#1 cells metastasized much less efficiently than that injected with MDA-MB-231-shCtrl cells, as illustrated by the bioluminescence imaging (Fig. 2i). Noticeably, histologic observation showed that mice bearing MDA-MB-231-shPRMT1#1 cells had no visible metastases, whereas macroscopic lung metastases were found in mice transplanted with MDA-MB-231-shCtrl cells (Fig. 2j). Similar results were observed in *in vivo* tail vein injection experiment with metastatic murine breast adenocarcinoma 4T1 cells (Supporting Information Fig. S2a–2e). Together, these data clearly demonstrate the critical role of PRMT1 in breast cancer metastasis.

### The PRMT1-induced EMT facilitated the acquisition of stem cell-like properties of breast cells

**Figure 3:**
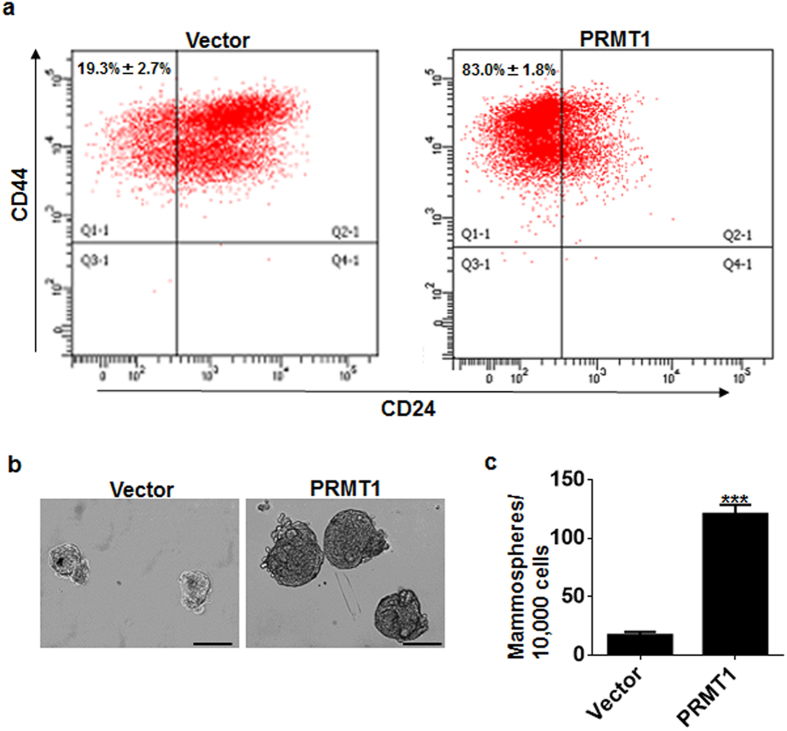
The PRMT1-induced EMT process generated stem cell-like phenotype in mammary epithelial cells. (**a**) Flow cytometric evaluation of CD44^high^/CD24^low^ subpopulation in MCF-10A-PRMT1 cells and in control vector cells. Percentages of mean CD44^high^/CD24^low^ subpopulation ± SD based on triplicate experiments. (**b**) Reprehensive images of mammospheres formation. Scale bar: 100 μm. (**c**) Quantification of mammosphere numbers formed from MCF-10A-PRMT1 cells and control vector cells in three independent experiments. Error bars, mean ± SD, ***P < 0.001.

Previous evidence suggests that both disseminated normal mammary epithelial cells and breast cancer cells undergoing the EMT process may acquire stem cell properties of CD44^high^/CD24^low^ antigen phenotype and mammosphere-formation ability^28^,^29^. To investigate whether PRMT1 can confer the MCF10A cells with the stem cell characteristics, we performed flow cytometry to sort the cells based on CD24/CD44 cell-surface markers to analysis cell populations with the CD44^high^/CD24^low^ expression pattern. We found that MCF10A-PRMT1 cells exhibited a significant increase (*P* = 0.0013) in the CD44^high^/CD24^low^ population compared with MCF10A-vector cells (Fig. 3a). Also, the mammosphere formation assays demonstrated an increase both in size and number of mammospheres in MCF10A-PRMT1 cells (Fig. 3b,c). Based on these results, we conclude that PRMT1 is able to induce MCF10A cells to develop stem cell characteristics and self-renewal capability.

### Depletion of PRMT1 arrested breast cancer cell growth in G1 tetraploidy and induced cellular senescence

**Figure 4:**
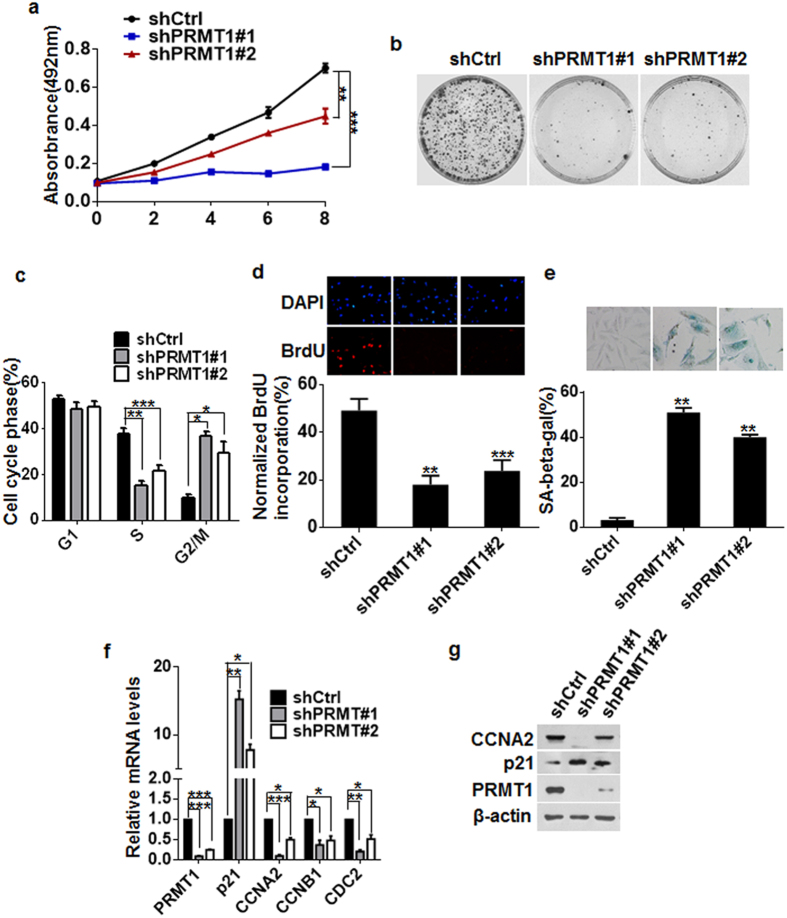
Knockdown of PRMT1 arrested breast cancer cell growth in G1 tetraploidy and induced cellular senescence. (**a**) Effect of PRMT1 knockdown on cell proliferation in MDA-MB-231 cells was assessed by MTT assays at different time points as indicated. Data are the mean ± SD of five replicates per experiment. (**b**) Cell proliferation was examined by colony formation assays in MDA-MB-231-shPRMT1#1/#2 and shCtrl cells. (**c**) Cell cycle analysis of MDA-MB-231-shPRMT1#1/#2 and shCtrl cells at day 4 after lentivirus infection. Percentages of subpopulation of cells at different cell cycle phases based on triplicate experiments. (**d**) Representative immunofluorescence images of BrdU incorporation in MDA-MB-231-shPRMT1#1/#2 and shCtrl cells. Lower panel represents the mean number of cells per field ± SD based on cell counts from five randomly chosen fields. (**e**) MDA-MB-231-shPRMT1#1/#2 and shCtrl cells were subjected to SA-β-Gal staining to determine the percentage of the senescent population (lower). Top panel represents images of SA-β-Gal staining. (**f**,**g**) Expression of p21 and G2/M associated proteins was analyzed by qRT-PCR (**f**) and western blotting (**g**). All experiments were repeated at least three times. Error bars, mean ± SD, *P < 0.05, **P < 0.01, ***P < 0.001.

An interesting phenomenon we have noticed in our experiments was that knockdown of PRMT1 in MDA-MB-231 cells not only attenuated the motility and invasion, but also led to a dramatic inhibition of cell growth (Fig. 4a,b). Meanwhile, we did not detect any enhancement in proliferative capacity in MCF10A cells overexpressing PRMT1 (Supporting Information Fig. S2). Analysis of DNA content by flow cytometry detected an elevated accumulation of MDA-MB-231-shPRMT1 cells in G2/M phase with 4N DNA content (Fig. 4c). Moreover, BrdU incorporation was remarkably diminished in PRMT1-depletion cells compared with control cells (Fig. 4d). Previous studies suggested that cells with 4N DNA content can also be arrested in G1 phase (G1 tetraploid cells)^30^, which emerged both in replicative and oncogenic Ras-induced senescence^31^,^32^. Senescent G1 tetraploid cells exhibit a dramatic downregulation of many G2/M genes, including CDC2, CCNB1 and CCNA2^33^,^34^. To test the hypothesis that MDA-MB-231-shPRMT1 cells have entered cellular senescence and undergone G1 arrest, we examined the activity of lysosomal senescence-associated β-galactosidase (SA-β-gal) and the expression of p21, CDC2, CCNB1 and CCNA2. The results revealed that both MDA-MB-231-shPRMT1 and MCF7-shPRMT1 cells showed a remarkably intensified SA-β-gal staining (Fig. 4e and Supporting Information Fig. S3a). Also, depletion of PRMT1 significantly increased the p21 expression, and reduced the CDC2, CCNB1 and CCNA2 expression at both mRNA and protein levels, in contrast to the control cells (Fig. 4f,g and Supporting Information Fig. S3b). Taken together, these data indicate that silencing of PRMT1 can lead to the mitotic errors and trigger the senescence of breast cancer cells, as a result of the downregulation of G2/M genes.

### PRMT1 activated ZEB1 expression through catalyzing the H4R3me2as modification at gene’s promoter

**Figure 5:**
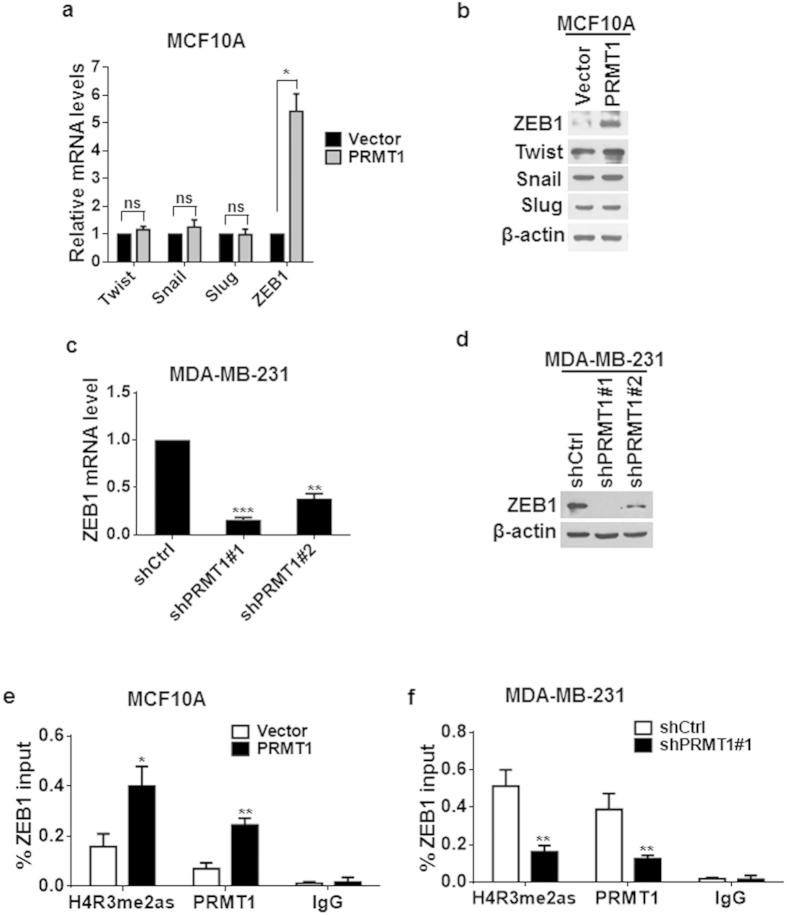
PRMT1 regulated ZEB1 expression through H4R3me2as. (**a**,**b**) Expression of known EMT inducers was assessed by qRT-PCR (**a**) and western blotting (**b**) in MCF10A-PRMT1 and in control vector cells. (**c**,**d**) Real-time RT-PCR and western blotting confirmation of ZEB1 knockdown efficiency in MDA-MB-231 cells. (**e**,**f**) Quantitative-ChIP experiments using anti-H4R3me2as and anti-PRMT1 to measure the levels of H4R3me2as and PRMT1 at the promoter of ZEB1 gene in MCF10A-PRMT1 cells (**e**) and MDA-MB-231-shPRMT7#1/#2 cells (**f**). All experiments were repeated at least three times. Error bars, mean ± SD, ns: non-significant, *P < 0.05, **P < 0.01, ***P < 0.001.

To understand the mechanism by which PRMT1 participates in breast cancer EMT and metastasis, we tested whether PRMT1 can activate the expression of the crucial EMT inducers. Interestingly, we found that only the ZEB1 expression was dramatically increased at both mRNA and protein levels, while the levels of other EMT inducers such as Twist, Snail and Slug, remained basically unchanged (Fig. 5a,b) in MCF10A-PRMT1 cells. Meanwhile, knockdown of PRMT1 in MDA-MB-231 cells remarkably decreased ZEB1 expression (Fig. 5c,d). Since the PRMT1-mediated histone modification of H4R3me2as is linked to transcriptional activation, we intended to determine whether this modification is associated with the active transcription of ZEB1. The results of quantitative chromatin immunoprecipitation (qChIP) assay revealed a prominent elevation in the enrichment of H4R3me2as, together with the presence of PRMT1, at the region between −327 and −179 bp of the ZEB1 promoter in MCF10A-PRMT1 cells (Fig. 5e). As expected, less occupancy of this ZEB1 promoter region by H4R3me2as and PRMT1 was observed in MDA-MB-231-shPRMT1 cells (Fig. 5f). These results clearly indicate that PRMT1 facilitated the ZEB1 transcriptional activation by enriching the H4R3me2as modification at the ZEB1 promoter.

### ZEB1 was essential for PRMT1-induced EMT, migration, invasion and acquisition of stem-cell-like properties

**Figure 6:**
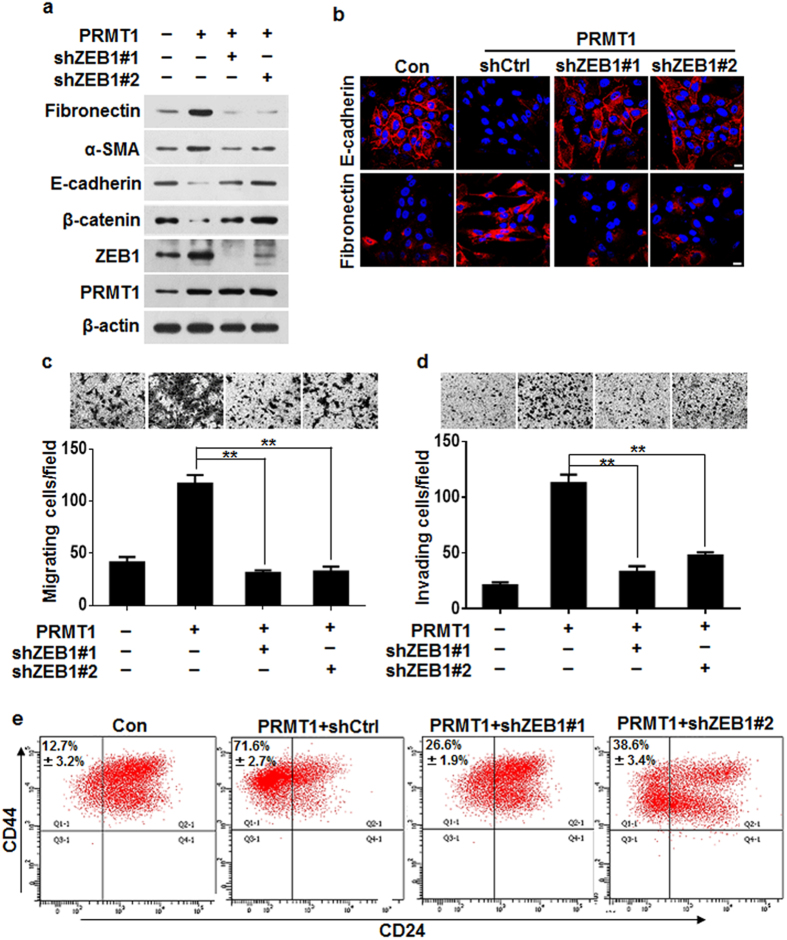
ZEB1 was essential for the PRMT1 function in MCF10A cells. (**a**) Silencing of ZEB1 decreased the expression of mesenchymal markers and restored the expression of epithelial markers in MCF10A-PRMT1 cells. (**b**) Representative immunofluorescence images of changes in epithelial and mesenchymal markers after silencing ZEB1 in MCF10A-PRMT1 cells. Scale bars: 10 μm. (**c**,**d**) Silencing of ZEB1 inhibited the PRMT1-driven Transwell migration and Matrigel invasion in MCF10A-PRMT1 cells. (**e**) Flow cytometric evaluation of CD44^high^/CD24^low^ subpopulation after ZEB1 knockdown in MCF-10A-PRMT1 cells. All experiments were repeated at least three times. Error bars, mean ± SD, *P < 0.05, **P < 0.01.

To evaluate whether ZEB1 eventually plays a role in the cellular events initiated by PRMT1, we virally transfected MCF10A-PRMT1 cells with two distinct ZEB1 shRNAs to specifically silence the ZEB1 expression. Indeed, we found that knockdown of ZEB1 counteracted the effects of PRMT1 overexpression. Specifically, ZEB1 suppression reduced the levels of mesenchymal cell markers fibronectin and α-SMA that were upregulated by PRMT1 expression (Fig. 6a). Meanwhile, silencing of ZEB1 markedly restored the levels of epithelial cell markers E-cadherin and β-catenin that were downregulated upon PRMT1 expression (Fig. 6a). Our immunofluorescence study further confirmed the changes of E-cadherin and fibronectin expression upon ZEB1 depletion (Fig. 6b). Meanwhile, silencing of ZEB1 strikingly attenuated the PRMT1-induced migratory and invasive abilities (Fig. 6c,d). Furthermore, we determined that loss of ZEB1 expression resulted in a considerable decrease of CD44^high^/CD24^low^ populations in MCF10A-PRMT1 cells (Fig. 6e), indicating that ZEB1 contributed critically to the acquisition of PRMT1-induced stem-cell-like property. These data are in support of our assumption that ZEB1 is necessary for PRMT1-induced EMT, migration, invasion and acquisition of stem-cell-like properties.

To summarize, we describe in this report a novel function of PRMT1 in modulating both EMT and cell growth in breast cancer cells. Specifically, PRMT1 is an important inducer for EMT, and is essential for the acquisition of stem cell properties and maintenance of proliferation in breast cancer cells. Loss of PRMT1 in aggressive breast cancer cells strongly blocks tumour metastasis *in vivo*, and inhibits breast cancer cells proliferation by inducing cellular senescence. Significantly, PRMT1 executes its function through catalyzing the H4R3me2as modification at a define region of ZEB1 promoter to activate the gene.

## Discussion

PRMT1 is the predominant member of the PRMTs family, responsible for at least 85% of all arginine methylation^35^. The alternative splicing of the human PRMT1 pre-mRNA generates seven protein isoforms (v1–v7) with a unique N-terminal sequence^36^. The PRMT1v1 and v2 mRNA expression was elevated in both breast cancer cell lines and in breast tumour tissues compared to their normal controls, and a strong correlation between PRMT1v1 and poor patient prognosis was established^25^,^37^. A pervious study emphasized that overexpression of PRMT1v2 isoform promoted motility and invasion in MCF7 breast cancer cells by phosphorylation-dependent downregulation of β-catenin, based on PRMT1v2′s cytoplasmic localization^38^. In this study, we focused on the function of the most abundant isoform PRMT1v1, which is predominantly localized to the nucleus. We found that PRMT1v1 was essential for the induction of EMT process, deciphering at least in part the action and involvement of PRMT1 in potentiating metastasis in malignant breast cancer. Moreover, PRMT1v1 was a potent regulator of E-cadherin, which is the most predominant hallmark of EMT in maintaining intact cell-cell contacts and preventing cell mobility and invasion. Presumably, the mechanisms by which PRMT1 affects breast cancer progression may vary between PRMT1v1 and PRMT1v2, regarding the distinct characteristics of enzymatic activity, substrate specificity and subcellular localization. These variations may account for the functional difference among the isoforms.

A major insight into the mechanistic action of PRMT1 made in this study is the discovery that the PRMT1-mediated histone H4R3me2as modification at the ZEB1 promoter region is responsible, at least in part, for the induction of EMT, and for the acquisition of stem cell properties and maintenance of proliferation in breast cancer cells. Human PRMT1 is an asymmetric arginine methyltransferase, and the H4R3me2as modification catalyzed by PRMT1 is a typical marker of active chromatin^22^. It has been known that, apart from histone H4, the substrates for PRMT1 cover a wide range of nonhistone proteins. A recent investigation showed that PRMT1 regulated EMT by methylating Twist1 at the arginine residue 34, which is required for E-cadherin repression in non-small cell lung cancer cells^39^. Our data revealed that, among the classic EMT-associated transcription factors (Twist, Snail, Slug and ZEB1) we tested, only the expression of ZEB1 was found to be regulated by PRMT1, and the PRMT1-mediated activation of ZEB1 was crucial for initiation of EMT process in breast cancer cells. Moreover, analysis of the primary amino acid sequences of the E-cadherin repressors revealed that both ZEB1 and ZEB2 harbored one potential PRMT methylation site (RG), whereas none was found in Snail nor in Slug^39^. Nevertheless, whether ZEB1 is a methylation substrate for PRMT1 needs to be further investigated. Besides, PRMT1 was recently identified to methylate Smad6 resulting in activation of TGFβ/Smad signaling pathway, which is also a potent inducer of EMT^40^. Apparently, multiple mechanisms may be involved in the initiation of EMT mediated by PRMT1.

Increasing evidence demonstrates that PRMT1 impacts crucial cellular pathways in cell cycle progression and cellular proliferation. Depletion of PRMT1 results in a marked attenuation in proliferation ability in a number of cancer cell lines, including osteosarcoma, breast, bladder and lung cancer cell lines^18^,^41^,^42^. However, the outcome of the anti-proliferation function of PRMT1 suppression has not been well identified. Results from this study first implicate that downregulation of PRMT1 with specific siRNAs induces senescence in breast cancer cells, as assessed by SA-β-gal staining (Fig. 4e and Supporting Information Fig. S3A). Consistent with the results from previous reports^39^,^41^, no typical sub-G1 peak, a common indicator of apoptosis, was observed in our experiments. Meanwhile, both MDA-MB-231-shPRMT1 and MCF7-shPRMT1 cells exhibited an increased expression of p21 (Fig. 4f,g). The cyclin dependent kinase inhibitors p21 and p16 are commonly upregulated to inhibit the Cdks, and thus to allow the accumulation of dephosphorylated pRb protein, resulting in growth arrest and senescence. Intriguingly, we found in this study that the senescence induced by PRMT1 inhibition was largely dependent on p21, but independent of p16. Indeed, both MCF7 cells (no detectable p16 expression) and MBA-MB-231 cells (deleted p16 expression) retained the ability to undergo senescence. Moreover, we also showed that the MDA-MB-231-shPRMT1 cells possessed the 4N DNA content, accompanied by a dramatic downregulation of several G2/M genes, such as cdc2, cyclin B1 and cyclin A2 (Fig. 4f,g and Supporting Information Fig. S3B). There have been indications that increased expression of p21 and formation of G1 tetraploidy senescence are often closely associated with senescence induced by cancer therapeutic agents in cancer cells^43^,^44^. Thus, information from this study and from others, implicates a new perspective that downregulation of PRMT1 may probably play an important role in trigger of senescence in cancer cells, although the detailed mechanisms of the action of PRMT1 in connection with p21 or mitotic errors remain to be elucidated.

It has been well established that EMT not only facilitates the metastatic dissemination of tumours, but also is associated with cancer therapy resistance^45^, stem cell traits^29^, apoptosis resistance^46^ and inhibition of senescence^10^. Several EMT-associated transcription factors like Snail, Twist and ZEB1, have attracted considerable research attention, due to their potentiality to contribute simultaneously to the inhibition of oncogene-induced senescence and to the promotion of EMT program^9^. An interesting finding arising from this study is that, ectopic expression of PRMT1 was able to induce EMT, whereas suppression of PRMT1 provoked senescence in breast cancer cells. We thus speculate that PRMT1 may probably function as an intermediate factor linking the two distinct cellular activities, i.e., EMT and cellular senescence, which play apparently distinct roles in tumorigenesis and cancer progression. Therefore, theoretically, PRMT1 can be targeted either to prevent cancer cell dissemination to develop metastatic lesions, or to eradicate existing metastatic cancer cells by induction of senescence. On the other hand, senescence leaves tumor cells alive and physiologically active in contrast to cell death. Senescent cells within the tumor can produce secreted factors with both tumor-promoting and tumor-suppressing activities^47^. Thus, such a treatment strategy targeted on PRMT1 might need to be combined with chemotherapy to prevent the activation of dormant metastases. However, the mechanistic insights of how PRMT1 overrides senescence and whether expression of ZEB1 can evade senescence induced by loss of PRMT1, etc., await for further investigations.

Cancer stem cells (CSCs) intrinsically contribute to dissemination for metastasis and therapeutic resistance, thus therapeutic strategies specifically targeting CSCs are rationally aimed to improve therapeutic outcome for cancer patients^44^. Data from our previous studies^45^,^48^,^49^ and from others^45^ have shown that EMT induces normal and neoplastic epithelial cells to generate CSC-like properties. In line with these results, we showed in this work that ectopic expression of PRMT1 in MCF-10A cells evidently elevated the percentage of cancer stem cell (CSC)-like cells that exhibit a CD44^high^/CD24^low^ antigenic phenotype and efficient mammosphere-formation ability. CSCs with these characteristics not only constitute a small minority of neoplastic cells within a tumour, and also contribute to seed new tumours at distant sites^50^. Significantly, our work suggests that PRMT1 is a new epigenetic regulator in generation of cancer sternness, and this finding provides a basis for the development of therapeutic strategy for highly aggressive and malignant breast cancers.

## Materials and Methods

### Cell cultures

MCF10A, MCF7, MDA-MB-231, MDA-MB-435, 4T1, BT549 and HEK293T cell lines were purchased from the American Type Culture Collection (ATCC, Manassas, VA, USA), and handled according to protocols provided by the ATCC. MCF10A cells were cultured as previously described^51^. MCF7, 4T1 and BT549 cells were cultured in RPMI-1640 medium (Sigma, St. Louis, MO, USA) supplemented with 10% FBS (ExCell Bio, Shanghai, China). MDA-MB-231 and MDA-MB-435 cells were cultured in L-15 medium (Sigma) with 10% FBS. HEK293T cells were cultured in DMEM medium (Sigma) containing 10% FBS. All the cell lines were grown at 37 °C with 5% CO_2_ except MDA-MB-231, which was cultured at 37 °C without CO_2_. All the cell lines were passaged in good condition.

### Plasmids and viral infection

The pcDNA3-PRMT1 expression vector was kindly provided by Dr. Akiyoshi Fukamizu (University of Tsukuba, Tsukuba, Japan). The lentiviral expression plasmid Pwpxld-PRMT1 was constructed from the cDNA sequence of pcDNA3-PRMT1 expression vector. The control, PRMT1 and ZEB1 short hairpin RNA plasmids were constructed in the pDSL-hpUGIP backbone. The lentivirus packaging vectors used were psPAX2 and pMD2.G. Generation of lentivirus in 293T cells and transfection of lentiviral constructs into recipient cell lines were performed following manufacturer’s instructions (Invitrogen). The sequences of shRNAs were described in Supplementary Table S1. Stable MCF10A-PRMT1 and PRMT1/ZEB1 knockdown cells were generated by lentivirus infection.

### RNA extraction, RT-PCR and real-time PCR analysis

Total RNA was extracted from cells using the Trizol reagent (TaKaRa, Dalian, China) following manufacturer’s instructions. The cDNA was generated using the Reverse Transcription System (Promega). Real-time PCR was carried out on a Roche LightCycler480 using SYBR Green Realtime PCR Master Mix (Roche). The β-actin was used as an internal control. The sequences of PCR primers were listed in Supplementary Table S1.

### Immunoblotting

Twenty μg of whole-cell lysate was loaded onto a 7.5–15% SDS-PAGE gel. Protein lysates were transferred to PVDF membrane (Millipore) and detected by ECL reagent (GE Healthcare, Buckinghamshire, UK). The following primary antibodies were used and diluted in 2% BSA/TBST: E-cadherin (BD Bioscience, #610181, 1:5000), fibronectin (BD Bioscience, #610077, 1:2000), vimentin (BD Bioscience, #550513, 1:8000), β-catenin (BD Biosciences, #610154, 1:2000), α-SMA (Sigma, #A5228, 1:3000), β-actin (Sigma, #A1978, 1:20000), Twist (Sigma, #sc-134136, 1:1000), MMP2 (GeneTex, #GTX104577, 1:1000), MMP9 (GeneTex, #GTX100458, 1:1000), PRMT1 (Millipore, #07-404, 1:5000), ZEB1 (Santa Cruz, #sc-25388, 1:500), p21 (Santa Cruz, #sc-756, 1:1000), cyclin A2 (Santa Cruz, #sc-751, 1:2000), Snail (Abcam, #ab63371, 1:2000), Slug (Abcam, #ab27568, 1:2000), and H4R3me2as (Active Motif, #39705, 1:1000).

### Immunofluorescence

Indirect immunolabeling was performed as described^52^. Cells were seeded on glass cover-slips in 12-well plates, left overnight before the treatment. Cells were fixed in 1% formaldehyde in culture medium for 10 min at 37 °C, then permeabilized with 0.2% Triton X-100 in PBS for 10min at 4 °C. Cells were then washed twice in PBS and blocked for 1 h with 5% BSA in PBS. Cells were incubated with primary antibodies at 4 °C overnight, washed three times in PBS and incubated with TRITC-conjugated secondary antibodies (Invitrogen) for 1 h at room temperature. Cell nuclei were counterstained with a 500 nM concentration of DAPI (Sigma). Photographs were taken under a confocal fluorescence microscope (Olympus Corporation, Tokyo, Japan) operating at 543nm laser spectrum.

### Wound healing, transwell migration and invasion assays

Experiments were performed basically as described previously^48^. For wound-healing assay, cells were seeded at a density of 1 × 10^6^ cells/well in 6-well plates. The progress of migration was photographed immediately and during 3 days after wounding (0/12/24/48/72 h) under an inverted microscope. The percentage of migration was calculated as the width of a scratch divided by the initial width of the same scratch times 100. At least five fields were analyzed for each scratch. 5 × 10^4^ cells were used for each migration assay, and 5 × 10^5^ cells were used for each invasion assay. After cells were incubated for 24h (migration) and 48 h (invasion), those adhered to the lower surface of the membrane were stained with 0.1% crystal violet (Sigma). Randomly selected fields were photographed and five fields per filter were counted using Image J software.

### Cell proliferation and SA-β-gal assays

MTT [3-(4, 5-dimethylthiazol-2-yl)-2, 5-diphenyl tetrazolium bromide] assay was used to assess cell proliferation at different time points (0/2/4/6/8 days). Cells were plated at 3 × 10^3^ cells/well on 96-well plates. For colony formation assays, 5 × 10^3^ cells were plated on 3.5 cm plates. Cells were fixed in 4% formaldehyde and stained with crystal violet after 10 days. BrdU incorporation assay was applied to measure the cell proliferation. BrdU pulses (10 nmol/ml, BD Biosciences) were performed in MDA-MB-231 cells for 6h and then labeled with TRITC-conjugated BrdU antibody (BD Biosciences). The stained cells were visualized under a fluorescence microscope, and at least 200 cells were counted for BrdU incorporation. SA-β-gal was detected according to the method described elsewhere^53^ with minor modifications. Cells were fixed in 2% formaldehyde in PBS, washed, and exposed overnight at 37 °C to a solution containing 1 mg/ml 5-bromo-4-chloro-3-indolyl-β-D-galactopyranoside (X-Gal), 5 mM potassium ferrocyanide, 5 mM potassium ferricyanide, 150 mM NaCl, 2 mM MgCl2, and 0.1 M phosphate buffer (pH 6.0). All the experiments were repeated three times, and one of the representative results was shown.

### Mammosphere formation assay

Experiments were performed as described previously^49^. 1 × 10^5^ cells were suspended in the ultralow attachment six-well plates (Corning) in DMEM/F12 medium (serum free) supplemented with 20 ml/ml B27 (Invitrogen), 20 ng/ml EGF (R&D), 20 ng/ml bFGF, 4 mg/mL insulin (Gibco), 4 mg/mL heparin, 1 mg/mL hydrocortisone (Sigma) and 0.4% BSA at 37 °C in 5% CO_2_. Fresh media was added every 3 days. Mammospheres over 50 μm in diameter were counted at day 10.

### Flow cytometry analysis

For cell cycle assay, ethanol-fixed MDA-MB-231 cells were analyzed for cellular DNA content after propidium iodide staining. For cancer stem cell assay, a total of 1 × 10^6^ cells were pretreated with 5% BSA blocking reagent to block non-specific antibody binding and incubated on ice protecting from light for 15 min with a panel of CD44-APC and CD24-PE (BD Biosciences) antibodies. Cells were then washed with PBS containing 2% BSA. The antibody-labeled cells were analyzed using a FACS Canto II flow cytometer (BD Biosciences, US). Data were analyzed with FACS Diva software (BD Biosciences, US). Control isotype-matched monoclonal antibodies were included to determine the level of background staining.

### Chromatin immunoprecipitation–quantitative PCR

The chromatin immunoprecipitation (ChIP) Kit was purchased from Millipore (Cat No. 17-10085) and ChIP experiments were carried out essentially in accordance with manufacturer’s guidelines. The ChIP Grade antibody against H4R3me2as was purchased from Active Motif (Cat No. 39705). Immnuoprecipitated DNA was amplified with the designated primers on the Roche LightCycler480. The primers for the ZEB1 promoter were: 5′-TCCAACTTTACCTTTCCAACTCCG-3′ (sense) and 5′-GCAACCGTGGGCACTGCTGAAT-3′ (antisense).

### *In vivo* mouse lung metastasis assays

MDA-MB-231-shPRMT1#1 cells, MDA-MB-231-shCtrl cells (2 × 10^6^) were injected into the tail veins of 5-week old female BALB/c nude mice. Two months later, the mice were sacrificed and lungs were fixed in formalin. For bioluminescence imaging, mice were injected i.p. with 150 mg/kg D-luciferin (GoldBio) 10 minutes before imaging. Following anesthesia, images were collected using the NightOWL LB 983 Spectrum Imaging System. The pDSL-shPRMT1#3 vector was used to knock down mouse PRMT1 expression. To simulate tumour cell metastasis, 4T1 cells stably expressed the control shRNA or the shPRMT1#3 (2 × 10^5^) were subcutaneously injected through tail vein of 10–14 weeks female BALB/c mice. Mice were sacrificed to measure the number of visible metastatic nodules 14 d after i.v. injection. All of the lung tissues were removed and fixed in 10% neutral buffered formalin for 48 h, dehydrated in 30% saccharose solution for 48h, embedded in Optimal Cutting Temperature (OCT) medium, and sectioned. Slides were stained with haematoxylin and eosin. Images were examined with an Olympus BX600 microscope (Olympus Corporation), and compiled with Adobe Photoshop and Illustrator CS5 (Adobe). All animal experiments were approved by the Animal Care Committee of the Northeast Normal University, China. The methods were carried out in accordance with the approved guidelines.

### Statistical analysis

Data were compiled from at least three independent, replicate experiments. Data are presented as mean ± SD. The paired Student’s *t*-test (two-tailed) was used to calculate the statistical significance of differences between groups. The *p* < 0.05 was considered statistically significant. Statistical analysis was carried out using the GraphPad Prism software (GraphPad Software, La Jolla, CA, USA).

## Additional Information

**How to cite this article**: Gao, Y. *et al.* The dual function of PRMT1 in modulating epithelial-mesenchymal transition and cellular senescence in breast cancer cells through regulation of ZEB1. *Sci. Rep.* **6**, 19874; doi: 10.1038/srep19874 (2016).

## Supplementary Material

Supplementary Information
