# Supplementary material for: The dual function of PRMT1 in modulating epithelial-mesenchymal transition and cellular senescence in breast cancer cells through regulation of ZEB1
Source: Sci Rep. 2016 Jan 27;6:19874. doi: 10.1038/srep19874 (PMC4728496; doi:10.1038/srep19874)
Supplement: Supplementary Information [file srep19874-s1.pdf]

Supplemental Information

The dual function of PRMT1 in modulating epithelial-mesenchymal transition and cellular senescence in breast cancer cells through regulation of ZEB1

Yanyan Gao, Yaping Zhao, Juechao Zhang, Yang Lu, Xin Liu, Pengyu Geng, Baiqu Huang, Yu Zhang & Jun Lu

Supplementary Figure S1

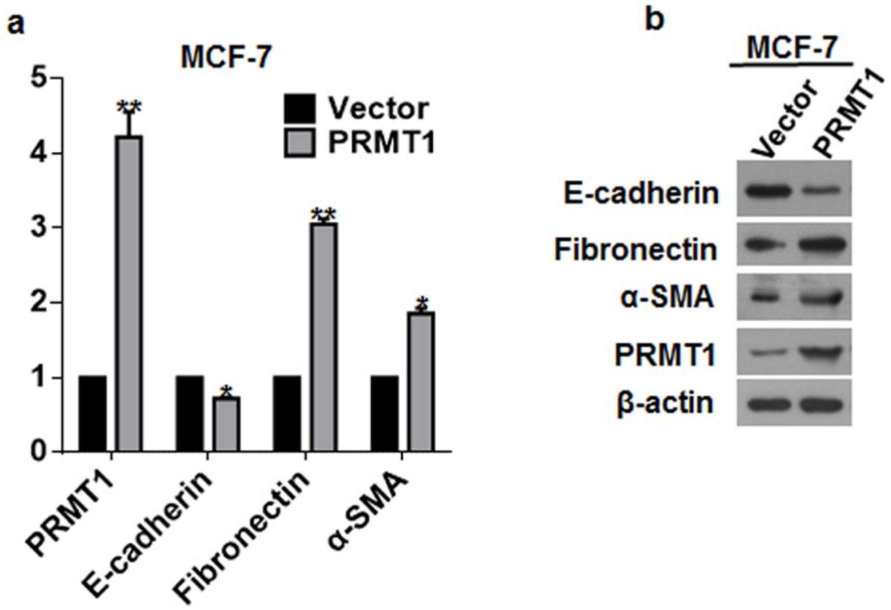

Supplementary Figure S1. Changes in mRNA and protein levels of the EMT marker genes upon ectopic expression of PRMT1 in MCF7 cells. a. The mRNA levels of PRMT1, E-cadherin, fibronectin and α-SMA were

assessed by real-time PCR in MCF7-PRMT1 cells. **b.** Western blots of the expression of PRMT1, E-cadherin, fibronectin and α-SMA in MCF7-PRMT1 cells. Error bars, mean ± SD, * P<0.05, ** P<0.01.

Supplementary Figure S2

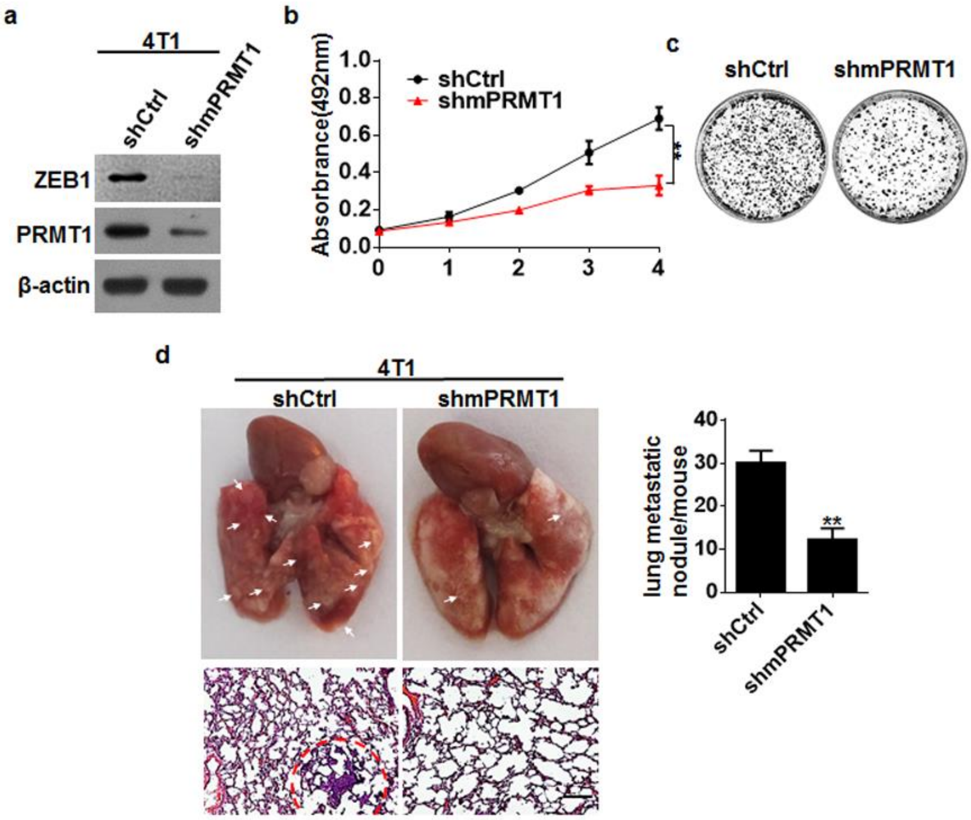

Supplementary Figure S2. PRMT1 knockdown on murine breast adenocarcinoma 4T1 cells inhibited cell proliferation, ZEB1 expression and the distant metastasis in the *in vivo* female BALB/c mice. a. Western blotting of mouse PRMT1 knockdown efficiency in 4T1-shmPRMT1, and expression level of ZEB1. **b.** MTT assay was used to assess the effect of PRMT1 knockdown on 4T1 cell growth at different time points as indicated.

Data are the mean ± SD based on five replicates per experiment. **c.** Cell proliferation was examined by colony formation assays in 4T1-shmPRMT1 and shCtrl cells. **d.** 4T1-shmPRMT1 cells and 4T1-shCtrl cells were subcutaneously injected into BALB/c female mice (n=6, for each experimental group), and 2 weeks later, mice were sacrificed, visible lung metastatic nodules per mouse were counted and are represented in the graph. Representative H&E stained lung sections are displayed in the lower panel. Scale bars: 100μm. Error bars, mean ± SD, ** P<0.01.

Supplementary Figure S3

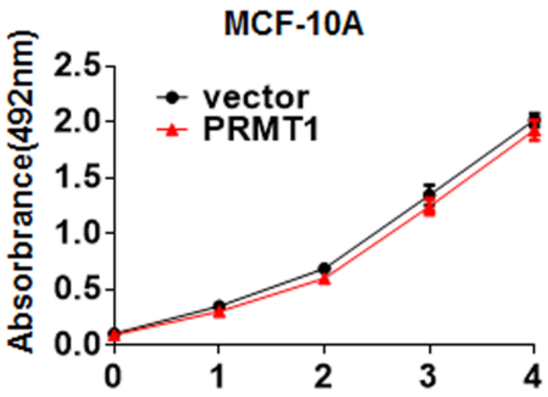

Supplementary Figure S3. Effects of PRMT1 overexpression on cell proliferation in MCF10A cells. MTT assay was used to assess the effect of PRMT1 overexpression on MCF10A cell growth at different time points as indicated. Data are the mean ± SD based on five replicates per experiment.

Supplementary Figure S4

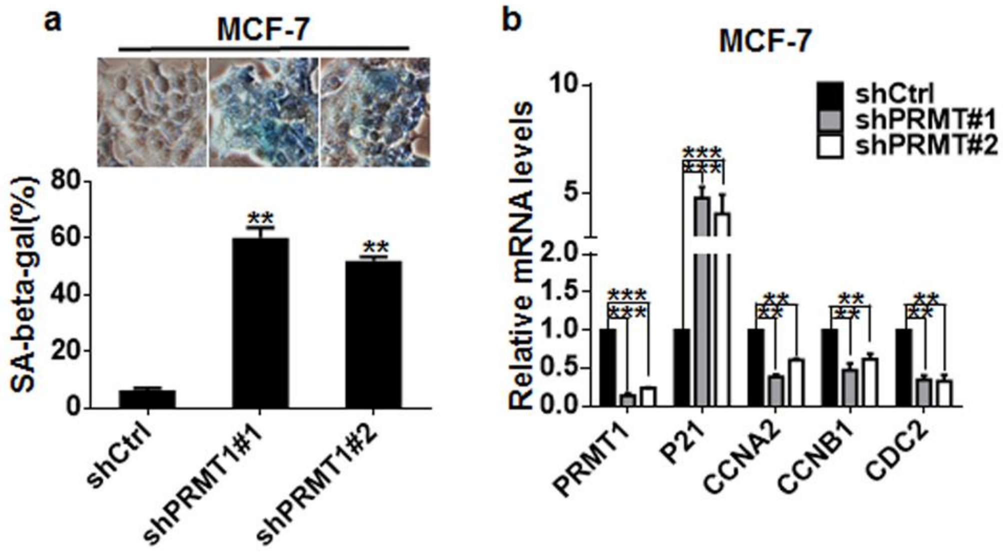

Supplementary Figure S4. PRMT1 knockdown induced cellular senescence in MCF7 cells. a. SA-β-Gal assay was used to determine the percentage of the senescent population in MCF7-shPRMT1#1/#2 and shCtrl cells (lower). Top panel shows the images of SA-β-Gal staining. **b.** Expression of p21 and G2/M-associated proteins was analyzed by qRT-PCR. All experiments were repeated at least three times. Error bars, mean ± SD, ***P*<0.01, ****P*<0.001.

Supplementary Table S1

Cloning Primers sequences	
shCtrl S	GATCCCCTTCTCCGAACGTGTCACGTTTCAAGA GAACGTGACACGTTCGGAGAATTTTTC
shCtrl AS	TCGAGAAAAATTCTCCGAACGTGTCACGTTCTC

	TTGAAACGTGACACGTTCGGAGAAGGG
shPRMT1#1 S	GATCCCCAGATTACTACTTTGACTCCTTCAAGAGAGGAGTCAAAGTAGTAATCTTTTTC
shPRMT1#1 AS	TCGAGAAAAAAGATTACTACTTTGACTCCTCTCTTGAAGGAGTCAAAGTAGTAATCTGGG
shPRMT1#2 S	GATCCCCGCGAGGAGATCTTCGGCACCATTCAAGAGATGGTGCCGAAGATCTCCTCGCTTTTTC
shPRMT1#2 AS	TCGAGAAAAAGCGAGGAGATCTTCGGCACCATCTCTTGAATGGTGCCGAAGATCTCCTCGCGGG
shPRMT1#3 S	GATCCCCGGACATGACATCCAAAGACTTCAAGAGAGTCTTTGGATGTCATGTCCTTTTTC
shPRMT1#3 AS	TCGAGAAAAAGGACATGACATCCAAAGACTCTCTTGAAGTCTTTGGATGTCATGTCCGGG
shZEB1#1 S	GATCCCCGCTCACACATAAGCAGTAAGATTCAAGAGATCTTACTGCTTATGTGTGAGCTTTTTC
shZEB1#1 AS	TCGAGAAAAAGCTCACACATAAGCAGTAAGATCTCTTGAATCTTACTGCTTATGTGTGAGCGGG
shZEB1#2 S	GATCCCCGCAACAGGGAGAATTATTAGATTCAAGAGATCTAATAATTCTCCCTGTTGCTTTTTC
shZEB1#2 AS	TCGAGAAAAAGCAACAGGGAGAATTATTAGATCTCTTGAATCTAATAATTCTCCCTGTTGCGGG
**PCR primers sequences**	

β-actin Forward	GAGCACAGAGCCTCGCCTTT
β-actin Reverse	ATCCTTCTGACCCATGCCCA
PRMT1 Forward	GATGCTGAAGGACGAGGTGC
PRMT1 Reverse	ACTCGATCCCGATGACCTTGCG
FN Forward	CAGTGGGAGACCTCGAGAAG
FN Reverse	TCCCTCGGAACATCAGAAAC
α-SMA Forward	CGTGTTGCCCCTGAAGAGCAT
α-SMA Reverse	ACCGCCTGGATAGCCACATACA
E-cad Forward	GACAACAAGCCCGAATT
E-cad Reverse	GGAAACTCTCTCGGTCCA
β-cat Forward	AAAATGGCAGTGCGTTTAG
β-cat Reverse	TTTGAAGGCAGTCTGTCGTA
Cyclin A2 Forward	CATTGGTCCCTCTTGATTATCC
Cyclin A2 Reverse	CACTCACTGGCTTTTCATCTTC
Cyclin B1 Forward	AAGAGCTTTAAACTTTGGTCTGGG
Cyclin B1 Reverse	CTTTGTAAGTCCTTGATTTACCATG
cdc2 Forward	TGGGGTCAGCTCGTTACTCA
cdc2 Reverse	CACTTCTGGCCACACTTCATT
p21 Forward	GGATGTCCGTCAGAACCC
p21 Reverse	GCTCCCAGGCGAAGTCA
Snail Forward	GCAAATACTGCAACAAGG
Snail Reverse	GCACTGGTACTTCTTGACA

Slug Forward	AGATGCATATTCGGACCCAC
Slug Reverse	CCTCATGTTTGTGCAGGAGA
Twist Forward	GGAGTCCGCAGTCTTACGAG
Twist Reverse	TCTGGAGGACCTGGTAGAGG
ZEB1 Forward	TGCACTGAGTGTGGAAAAGC
ZEB1 Reverse	TGGTGATGCTGAAAGAGACG
